# HPV genotype determination and E6/E7 mRNA detection for management of HPV positive women

**DOI:** 10.1186/s12985-018-0957-z

**Published:** 2018-03-27

**Authors:** Maria Teresa Bruno, Martina Ferrara, Valentina Fava, Agnese Rapisarda, Angela Coco

**Affiliations:** Department of General Surgery and Medical Surgical Specialties, Gynecological Clinic of the University of Catania. Policlinico, Via Santa Sofia 78, 95124 Catania, Italy

## Abstract

**Background:**

Clinical management of HPV positive women is difficult since many of the infections, including high-risk oncogene genotypes (hr-HPV), are transient. Therefore only a limited number of patients have a high-grade lesion and sending all HPV positive women for colposcopy would only increase costs and unnecessary treatment, with serious psychological consequences for patients. The need has emerged to identify other HPV related markers able to correctly detect women with a high-risk of developing high-grade lesions. Genotyping and the search for E6/E7 mRNA are among the possible candidates.

**Methods:**

The study was carried out by means of an observational analysis of the data relative to 674 HR-HPV positive women who we had observed from January 2013 to June 2015; the data had been gathered in a database at the HPV Center of the University Hospital of Catania, Italy.

Women were considered eligible for this study if the following data was present in the database: Pap TEST, histologic evaluation, HPV TEST and E6/E7 mRNA detection.

We calculated the Odds Ratio (OR) of woman who were mRNA positive, with CIN2+ lesions, and Odds Ratio of HPV16 positive women.

**Results:**

Transcripts were detected in 23.6% (69/292) of the women with CIN1 and in 97.2% (210/220) of those with CIN2 + .

Regarding genotyping, the 81,8% (180/220) of the women with CIN2+ had genotype 16, while only 18.1% (40/220) had genotype 18, 31, 33, 45.

We calculated the OR in the group of HPV16 women with CIN2+ (OR = 4.62; 95% CI = 3.13 to 6.82), this value increased (OR = 106.12; 95% CI = 53.71 to 209.69) in women with CIN2+ and positive mRNA.

**Discussion:**

The presence of the HPV16 genotype in our study was associated with a risk 5 times greater of developing a high-grade lesion (CIN2+) (OR = 4.62 95% CI:3.13–6.82); this supports the hypothesis that it would be opportune to have targeted protocols for the management of HPV 16 positive women. The results showed that there was an association between E6/E7 mRNA expression and histology (OR = 106.12; 95% CI = 53.71 to 209.69). The E6/E7 mRNA test showed a higher prevalence of E6 and E7 transcripts in patients with higher-grade lesions.

**Conclusion:**

The results of this study suggest that the HPV genotype determination and E6/E7 mRNA detection would find an important application for management of HPV positive women.

## Background

Numerous studies carried out in various countries on a large number of women have confirmed the greater sensitivity of the HPV test to detect CIN2+ lesions with respect to traditional cytology [1–3] increasing the screening gap from 3 to 5 years for those patients who are negative at the HPV test. These results encouraged us to set out new Italian guidelines for the screening of cervical carcinoma in which the HPV DNA test is considered the gold standard for women > 35 years, and the Pap test for women in the 25–34 year range.

The problem arises with HPV positive woman; how best to manage them if only a limited number have a high-grade lesion and sending all HPV positive women for a colposcopy would only increase costs and unnecessary treatment, with severe psychological consequences for the patients. Over the last 10 years many researchers have tried to identify, among the patients positive at the HPV test, those at high-risk of neoplastic progression (CIN2+ lesions). In fact, with the simple negative/positive result to the HR-HPV virus it is possible to evaluate the prognostic role of various markers; this knowledge can help clinicians manage HPV positive cases better.

In the light of the numerous studies, it appears that genotype determination and the search for E6/E7 mRNA are still two markers that help clinicians in the management of woman with an anomalous Pap test.

### Genotype determination

Scientific evidence has shown that the evolution to CIN2+ lesions is greater with persistent HR-HPV infections [4, 5], therefore, knowing the genotype can improve the course of patient treatment. The most frequent genotype in all global case reports of CIN2+ lesions is HPV16 (51.2%) [6]. Its persistence in a female carrier makes her a woman at risk.

Knowing the incidence of the genotypes in the population can be fundamentally important as regards the preparation of prophylactic vaccines, which could vary their multivalency based on the most frequent genotypes in that given area [7].

### Positivity for E6/E7 mRNA

Many studies associate E6/E7 mRNA positivity with an integration of the progression of carcinogenesis, revealing an association between degree of integration, and histological, and cytological lesion severity [8–10]. However, the role of the viral genome integration process in the genome of the host cell is still unclear, in as much as other studies have not revealed any correlation with lesion severity [11, 12].

In our retrospective, observational study we wanted to investigate the positive HPV population that came to our center for secondary screening. We evaluated the incidence, the frequency of the various genotypes, their relationship with the histological lesions, and the capacity of the mRNA test to identify women at risk of CIN2+ lesions.

## Methods

### Study population

The study was carried out by means of an observational analysis of the data relative to 674 HR-HPV positive women who we had observed from January 2013 to June 2015; the data had been gathered in a database at the HPV Center of the University Hospital of Catania, Italy.

Women were considered eligible for this study if they satisfied the following criteria:

- □ Positive for HPV genotype 16, 18, 31, 33, 45;
- □ not pregnant;
- □ no evidence of any immunodeficiency;
- □ no history of therapy for neoplasms.

The average age of the women was 36.6 ± 9.5 years (range 22–62). To include a patient in the retrospective study, the following data had to be present in the database: pap smears, histologic evaluation, HPV TEST and mRNA test.

The smears, by conventional cytology, were classified according to the 2001 Bethesda System Criteria [13]: negative for intraepithelial lesions or malignancy (NILM), atypical squamous cells of unknown significance (ASCUS), (ASCH), low-grade squamous intraepithelial lesion (LSIL), high-grade squamous intraepithelial lesion (HSIL) and squamous cervical carcinoma (SCC).

If patients were HPV DNA positive for at least one of the 5 HR HPV types (HPV 16, 18, 31, 33, 45) they were analyzed for the expression of viral oncogenes E6 and E7, looking for the mRNA by the NucliSens EasyQ HPV assay method (bioMerieux).

Analyses of the samples were performed by the Virology Laboratory at the University Hospital of Catania, Italy.

Histologic evaluation was performed with specimens collected by a colposcopy-directed biopsy and/or cone specimens collected by the loop excision procedure. The histological slides were diagnosed according to the WHO classification as CIN2+ for all the cases of CIN2, CIN3, and SCC lesions.

The women who showed progression to CIN2+ underwent large loop excision of the transformation zone (LLETZ).

### HPV DNA detection

Exo-endocervical cytology samples were collected in ThinPrep solution for total nucleic acid extraction (DNA and RNA) for detection and genotyping of viral DNA by means of gene amplification. This was followed by hybridization with genotype-specific probes able to identify most of the genital region HPV types.

The automated DNA extraction was carried out with 1 ml sample on the NucliSenseasyMAG system (bioMérieux SA, Marcy l’Etoile, France) following the manufacturer’s HPV 1.1 protocol, with a 55 μl final elution volume.

### HPV E6/E7 mRNA detection

A total of 5 ml of Preserv Cytsolution was used for the detection of E6/E7 mRNA of HPV types 16, 18, 31, 33, and 45 by means of the PreTect HPV-Proofer Kit (referred to as mRNA test) (Biomérieux, Italy) within 14 days of sample collection, according to the manufacturer’s instructions. mRNA was extracted from cervical samples using NucliSENS mini MAGMagnetic Extraction (Biomérieux, Italy). The PreTect HPV-Proofer technology uses an isothermal nucleic acid sequence–based amplification (NASBA) that amplifies mRNA in a DNA background, detecting and genotyping HPV transcripts in the same reaction.

The mRNA testing was performed according to the manufacturer’s instructions NucliSENS Easy Q HPV (bioMérieux, Italy) and in accordance with national guidelines for HPV testing.

### Statistical analysis

Statistical analysis was carried out using SPSS for Windows (version 15.0, SPSS, Chicago, IL, USA). Descriptive statistics were expressed by frequency, arithmetic mean, standard deviation (SD) and percentages. We calculated the Odds Ratio (OR) of women with mRNA positive, with CIN2+ lesions, the Odds Ratio (OR) of women with HPV16.

## Results

The 674 HR-HPV positive women underwent biopsy; Table 1 shows the histological results.

**Table 1:** Histologic findings of the 674 positive HPV women enrolled

Histology	N°	%
No Atypia	162	24
CIN1	292	43.3
CIN2+	220	32.6
total	674	

The results of HPV test are shown in Table 2. Among the histologic lesions the most frequent genotype found was HPV16 (404 cases) with a total frequency of 59.9% (Table. 2). Its frequency shows a clear trend, increasing with lesion increase, with a percentage of 23.2% in negative histology, 32.1% in CIN1, and 44.5% in CIN2+. The second most frequently detected genotype was HPV31 with a total incidence of 17.2%. The lesions in which it was most present were CIN1 48.2% and CIN2+ 17.2%; it was present in 5 CIN3 lesions. The genotype HPV18 had a total frequency of 14.5% with an incidence of 65.3% found in CIN1, 14.2% in CIN2+, our study revealed no neoplasias. The third genotype in order of frequency was HPV33 (5.19%) that was responsible for two cases of CIN2 and two cases of CIN3. The genotype 45 was not frequent in our case study with a total frequency of 4.45% and was responsible for only one case of CIN3.

**Table 2:** CIN1 and CIN2+ (percentage) in the HPV genotypes 16, 18, 31, 33 and 45

HPV	N°	No atypia	CIN1	CIN2+
HPV16	404 (59.9%)	94 (23.3%)	130 (33.2%)	180 (44.6%)
HPV31	116 (17.2%)	40 (34.5%)	56 (48.3%)	20 (17.2%)
HPV18	98 (14.5%)	20 (20.4%)	64 (65.3%)	14(14.3%)
HPV33	35 (5.2%)	5 (14.6%)	26 (74.3%)	4 (11.4%)
HPV45	21 (3.1%)	3 (14.3%)	16 (76.2%)	2 (9.5%)
total	674	162	292	220

The results of E6/E7 mRNA expression are shown in Table 3. Transcripts were detected in 3.7% (6/162) of no atypia, in 23.6% (69/292) of those with CIN1 and in 95.4% (210/220) of those with CIN2 + .

**Table 3:** mRNA positivity with genotypes and histology evaluation

mRNA +						
Histology						
Genotype	No atypia	%	CIN 1	%	CIN 2	%
HPV 16	3	50.0	42	60.9	178	84.8
HPV 18	2	33.3	9	13.0	10	4.8
HPV 31	1	16.7	8	11.6	18	8.6
HPV 33	0	0	5	7.2	3	1.4
HPV 45	0	0	5	7.2	1	0.5

Regarding genotyping, the 81.8% (180/220) of the women with CIN2+ had genotype 16, while only 18.1% (40/220) had genotype 18, 31, 33 and 45 (Table 4).

**Table 4:** CIN2+ in genotypes 16, 18, 31, 33 and 45

HPV	CIN2+	%
16	180	81.8
31	20	9.1
18	14	6.4
33	4	1.8
45	2	0.9

We calculated the Odds Ratio in the group of HPV16 women with CIN2+ (OR = 4.62; 95% CI = 3.13 to 6.82), this value increased (OR = 106.12; 95% CI = 53.71 to 209.69) in women with CIN2+ and positive mRNA.

## Discussion

The onset of a carcinoma is not due to the presence of the virus but its integration [14]. On this is based the development of the method that evaluates the presence of mRNA, due to the fact that, in cancerogenesis, the over-expression of the viral oncogenes E6 and E7 is essential both for the start and the maintenance of the transformed phenotype induced by HR-HPV. The detection of the mRNA of the oncogenes E6/E7 could be a better marker to predict the development of cancer with respect to the detection of HPV DNA [15] and can distinguish the transient infections from persistent ones [16].

The method is a qualitative assay based on the NASBA method that detects the mRNA of 5 HR-HPV (16, 18, 31, 33, 45); it has a cellularity check that targets the human U1A gene. Studies reported in the literature show its greater specificity with respect to the Aptima test and HC2, but it shows a lower sensitivity for the detection of CIN2+ lesions [17].

The rationale behind the mRNA test is convincing, with a good sensitivity and greater specificity. Among the various problems to be overcome there are: sample storage (mRNA is very unstable and the possible use of this method in screening protocols requires efficient collecting systems to avoid degradation); the management of the woman with a negative mRNA test but a positive HPV DNA test.

The growing interest in molecular studies has led various authors to compare the performance of the HPV DNA test with HPV mRNA test, evaluating the diagnostic accuracy in identifying high-grade lesions [18–20].

From a recent systematic review [21], it was found that the HPV mRNA test gives fewer false positives than the HPV DNA test, with consequent greater specificity.

However, very few studies have verified the role of the mRNA test in predicting the development of a high-grade lesion during follow-up. The results showed that there was an association between E6/E7 mRNA expression and histology evaluation.

In our study, the mRNA test was negative in 4.5% (10/220) of CIN2+ lesions. Taking into account that some CIN2+ lesions may spontaneously regress, mainly when they are CIN2 [22], we could hypothesize that the pre-invasive lesions that are negative for E6/E7 mRNA could be more susceptible to regression. Benevolo [23] (2011) found the mRNA test was negative in 43% of histologically confirmed high-grade lesions. Even the Ronco [24] study (2008), and the Moscicki [25] study (2010) showed that a regression of CIN2+ is frequent mostly in younger HPV positive women**.** However, it cannot be verified whether an mRNA test negative in CIN2+ cases could be associated with a higher probability of regression, since CIN2+ women, according to local on-going protocols, usually undergo excisional treatment.

Cattani P. et al. [26], (2009) on 180 women, evaluated the presence of HPV DNA (HC II) and mRNA (NucliSENSE) with colposcopy and histology. The authors suggested that mRNA assays could be more powerful than DNA testing for predicting the risk of progression and offered a strong potential as a tool for triage and patient follow-up.

## Conclusions

Genotype determination for woman with alterations on the Pap test is fundamental for the following reasons as it: detects the HPV genotype responsible for infection; detects multiple infections, which can be a marker for lesion progression; and discriminates between new infections and pre-existent infections. The presence of HPV16 genotype in our study was associated with a risk 5 times greater of developing a high-grade lesion (CIN2+) (OR = 4.62 95% CI:3.13–6.82), supporting the hypothesis that it would be opportune to have targeted protocols for the management of HPV16 positive woman; in fact, according to the latest up-date of the guidelines of the American Society for Colposcopy and Cervical Pathology (ASCCP), since the HPV16 virus is much less common than CIN1 lesions, with respect to CIN3 lesions, women positive for HPV 16, even with negative cytology, (women 30 and older who have discordant results) should be sent for colposcopy [27]. From a prognostic point of view, the positivity of E6/E7 mRNA may be associated with a higher risk of having a CIN2+ lesion (OR = 106.12; 95% CI = 53.71 to 209.69). These data are, in part, in agreement with what has been found by other authors. Burger et al. [21] (2011) performed a systematic review to determine the performance of HPV mRNA testing compared to DNA testing using CIN2+ as the target condition. The results of this study, considering that there can be cases in which the integrated viral genome is no longer visible to the DNA test but can be detected by means of the mRNA detection of the viral oncoproteine [28], suggest that the detection of mRNA of the viral oncoproteine E6/E7 (especially with Aptima, 14 genotypes), used in conjunction with the pap test, would find an important application in HPV infection screening.

## Abbreviations

ASCUS (ASCH): Atypical squamous cells of unknown significance
CIN: Cervical intraepithelial lesion
CIN2 +: All the cases of CIN2, CIN3, SCC lesions
HPV: Human Papilloma Virus
HR-HPV: High risk-HPV
HSIL: High-grade squamous intraepithelial lesion
LSIL: Low-grade squamous intraepithelial lesion
NILM: Negative for intraepithelial lesions or malignancy
OR: Odds Ratio
SCC: Squamous cervical carcinoma

## References

[1] Cuzick J, Szarewski A, Cubie H, Hulman G, Kitchener H, Luesley D, McGoogan E, Menon U, Terry G, Edwards R, Brooks C, Desai M, Gie C, Ho L, Jacobs IO, Sottaceti C, Sasieni P (2003). Management of women who test positive for high-risk types of human papillomavirus: the HART study. Lancet.

[2] Cuzick J, Clavel C, Petry KU, Meijer CJ, Hoyer H, Ratnam S, Szarewski A, Birembaut P, Kulasingam S, Sasieni P, Iftner T (2006). Overview of the European and North American studies on HPV testing in primary cervical cancer screening. Int J Cancer.

[3] Ronco G, Biggeri A, Confortini M, Rossi PG, Naldoni C, Segnan N, Sideri M, Zappa M, Zorzi M. HPV DNA based primary screening for cervical cancer precursors Epidemiologia & Prevenzione. Epidemiol Prev. 2012;36:1–72. 22828243

[4] Muñoz N, Bosch FX, Castellsagué X, Díaz M, De Sanjose S, Hammouda D, Shah KV, Meijer CJ (2004). Against which human papillomavirus types shall we vaccinate and screen? The international perspective. Int J Cancer.

[5] Saslow D, Solomon D, Lawson HW, Killackey M, Kulasingam S, Cain J, Garcia FA, Moriarty A, Waxman A, Wilbur D, Wentzensen N, Downs L, Spitzer M, Moscicki AB, Franco EL, Stoler MH, Schiffman M, Castle PE, Myers ER (2012). American Cancer Society, American Society for Colposcopy and Cervical Pathology, and American Society for Clinical Pathology Screening Guidelines for the prevention and early detection of cervical Cancer. CA Cancer J Clin.

[6] Sjoeborg KD, Trope A, Lie AK, Jonassen CM, Steinbakk M, Hansen M, Jacobsen MB, Cuschieri K, Eskild A (2010). HPV genotype distribution according to severity of cervical neoplasia. Gynecol Oncol.

[7] García-Espinosa B, Moro-Rodríguez E, Alvarez-Fernández E (2012). Genotype distribution of human papillomavirus (HPV) in histological sections of cervical intraepithelial neoplasia and invasive cervical carcinoma in Madrid, Spain. BMC Cancer.

[8] Peitsaro P, Johansson B, Syrjänen S (2002). Integrated human papillomavirus type 16 is frequently found in cervical cancer precursors as demonstrated by a novel quantitative real-time PCR technique. J Clin Microbiol.

[9] Zhang D, Zhang Q, Zhou L, Huo L, Zhang Y, Shen Z, Zhu Y (2010). Comparison of prevalence, viral load, physical status and expression of human papillomavirus-16, −18 and −58 in esophageal and cervical cancer: a case-control study. BMC Cancer.

[10] Saunier M, Monnier-Benoit S, Mauny F, Dalstein V, Briolat J, Riethmuller D, Kantelip B, Schwarz E, Mougin C, Pretet JL (2008). Analysis of human papillomavirus type 16 (HPV16) DNA load and physical state for identification of HPV16-infected women with high-grade lesions or cervical carcinoma. J Clin Microbiol.

[11] Cheung LK, Cheung TH, Ng CWY, Yu MY, Wong MCS, Siu SSN, Yim SF, Chan PKS. Analysis of human papillomavirus type 18 load and integration status from low-grade cervical lesion to invasive cervical Cancer. J Clin Microbiol 2009; 47: 287–293. 10.1128/JCM.01531-08 PMC2643667 19036939

[12] Azizi N, Brazete J, Hankins C, Soldi D, Fontaine J, Koushik A, Rachlis A, Pourreaux K, Ferenczy A, Franco E, Coutlée F (2008). Canadian Women’s HIV study group. Influence of human papillomavirus type 16 (HPV-16) E2 polymorphism on quantification of HPV-16 episomal and integrated DNA in cervicovaginal lavages from women with cervical intraepithelial neoplasia. J Gen Virol.

[13] Solomon D, Davey D, Kurman R, Moriarty A, O'Connor D, Rapace M, Raab S, Sherman M, Wilbur D, Wright T, Giovani N (2002). Forum Group Members, Bethesda 2001 Workshop. The 2001 Bethesda system: terminology for reporting results of cervical cytology. JAMA.

[14] Corden SA, Sant-Cassia LJ, Easton AJ, Morris AG (1999). The integration of HPV-18 DNA in cervical carcinoma. J Clin Pathol Mol Pathol.

[15] Abreu ALP, Souza RP, Gimenes F, Consolaro MEL (2012). A review of methods for detect human papillomavirus infection. Virol J.

[16] Clad A, Reuschenbach M, Weinschenk J, Grote R, Rahmsdorf J, Freudenberg N (2011). Performance of the Aptima high-risk human papillomavirus mRNA assay in a referral population in comparison with hybrid capture 2 and cytology. J Clin Microbiol.

[17] Ratnam S, Coutlee F, Fontaine D, Bentley J, Escott N, Ghatage P, Gadag V, Holloway G, Bartellas E, Kum N, Giede C, Lear A (2011). Aptima HPV E6/E7 mRNA Test Is as sensitive as hybrid capture 2 assay but more specific at detecting cervical Precancer and Cancer. J Clin Microbiol.

[18] Varnai AD, Bollmann M, Bankfalvi A, Speich N, Schmitt C, Griefingholt H, Kovács K, Klozoris C, Bollmann R (2008). Predictive testing of early cervical pre-cancer by detecting human papillomavirus E6/E7 mRNA in cervical cytologies up to high-grade squamous intraepithelial lesions: diagnostic and prognostic implications. Oncol Rep.

[19] Cuschieri KS, Whitley MJ, Cubie HA (2004). Human papillomavirus type specific DNA and RNA persistence implications for cervical disease progression and monitoring. J Med Virol.

[20] Molden T, Kraus T, Karlsen F, Skomedal H, Nygard JF, Hagmar B (2005). Comparison of human papillomavirus messenger RNA and DNA detection: a cross-sectional study of 4, 136 women 30 years of age with a 2-year follow-up of high-grade squamous intraepithelial lesion. Cancer Epidemiol Biomark Prev.

[21] Burger EA, Kornør H, Klemp M, Lauvrak V, Kristiansen IS (2011). HPV mRNA tests for the detection of cervical intraepithelial neoplasia: a systematic review. Gynecol Oncol.

[22] Ronco G, Giorgi-Rossi P, Carozzi F, Dalla Palma P, Del Mistro A, De Marco L, De Lillo M, Naldoni C, Pierotti P, Rizzolo R, Segnan N, Schincaglia P, Zorzi M, Confortini M, Cuzick J (2006). New technologies for cervical cancer screening working group the new Technologies for Cervical Cancer screening (NTCC) working group. Human papillomavirus testing and liquid-based cytology in primary screening of women younger than 35 years: results at recruitment for a randomized controlled trial. Lancet Oncol.

[23] Benevolo M, Terrenato I, Mottolese M, Marandino F, Carosi M, Rollo F, Ronchetti L, Muti P, Mariani L, Sindico S, Vocaturo G, Vocaturo A (2011). Diagnostic and prognostic validity of the human papillomavirus E6/E7 mRNA test in cervical cytological of HC2-positive patients. Cancer Causes Control.

[24] Ronco G, Giorgi-Rossi P, Carozzi F, Confortini M, Dalla Palma P, Del Mistro A, Gillio-Tos A, Minucci D, Naldoni C, Rizzolo R, Schincaglia P, Volante R, Zappa M, Zorzi M, Cuzick J, Segnan N (2008). New technologies for cervical cancer screening working group. Results at recruitment from a randomized controlled trial comparing human papillomavirus testing alone with conventional cytology as the primary cervical cancer screening test. J Natl Cancer Inst.

[25] Moscicki AB, Ma Y, Wibbelsman C, Darragh TM, Powers A, Farhat S, Shiboski S (2010). Rate of and risks for regression of cervical intraepithelial neoplasia 2 in adolescents and young women. Obstet Gynecol.

[26] Cattani P, Zannoni GF, Ricci C, D'Onghia S, Trivellizzi IN, Di Franco A, Vellone VG, Durante M, Fadda G, Scambia G, Capelli G, De Vincenzo R (2009). Clinical performance of human papillomavirus E6 and E7 mRNA testing for high-grade lesions of the cervix. J Clin Microbiol.

[27] Massad LS, Einstein MH, Huh WK, Katki HA, Kinney WK, Schiffman M, Solomon D, Wentzensen N, Lawson HW (2013). 2012 updated consensus guidelines for the Management of Abnormal Cervical Cancer Screening Tests and Cancer Precursors. J Lower Genital Tract Dis.

[28] Sørbye SW, Fismen S, Gutteberg TJ, Mortensen ES, Skjeldestad FE (2014). HPV mRNA is more specific than HPV DNA in triage of women with minor cervical lesions. PLoSOne.

